# Laser thermal ablation to treat a recurrent soft-tissue sarcoma of the leg: a case report

**DOI:** 10.3332/ecancer.2019.908

**Published:** 2019-03-05

**Authors:** Chiara De Angelis, Paolo Della Vigna, Gianluca Maria Varano, Giovanni Mauri

**Affiliations:** 1 Division of Radiology, IEO, European Institute of Oncology IRCCS, via Ripamonti 435, 20141 Milan, Italy; 2 Division of Interventional Radiology, IEO, European Institute of Oncology IRCCS, via Ripamonti 435, 20141 Milan, Italy

**Keywords:** sarcoma, laser ablation, interventional radiology, amputation

## Abstract

We present the case of a 52-year-old male patient with recurrence of a soft-tissue sarcoma of the left leg treated with percutaneous laser ablation. The patient received the diagnosis of sarcoma for the first time in 2011; further local recurrences and a pulmonary metastatic spread occurred during follow-up, so the patient has been treated several times with chemotherapy, limb-sparing surgery and radiotherapy. In September 2017, a new local recurrence of sarcoma occurred, for which limb amputation was proposed but refused by the patient. Laser ablation with ultrasound guidance was performed, with complete ablation at 6 months and limb salvage.

## Introduction

Sarcomas represent a heterogeneous group of about 100 subtypes of soft-tissue and bone tumours of mesenchymal origin [1]. They are rare in the adult population, accounting for approximately 1% of all newly diagnosed malignancies annually [1–3], while they are more frequent in the paediatric population, accounting for 15% of all the tumours in this age group [3].

The first-line treatment for early stage (non-metastatic) sarcomas is surgery, which must be carried out as early as possible to increase healing probability [1–4]. In fact, if surgically removed, sarcomas can be cured [1].

Even after a complete remission following the primary treatment, 30%–35% of bone sarcoma patients and about 50% of soft tissue sarcoma patients experience local or systemic relapse [3, 4]. The complete removal of all tumours, irrespective of local or systemic recurrence, is advocated to achieve long-term survival [3].

Nevertheless, not all patients benefit from surgical treatment and unfortunately, many patients are metastatic at the onset of the disease while more than 50% of patients develop metastases during the course of their disease [1, 3]. Lungs are the predominating site of metastases and the exclusive site in over 50% of patients at the first metastatic event [1, 3, 5]. In patients with metastatic disease, the standard treatment is neoadjuvant chemotherapy and radiotherapy; chemotherapy generally prolongs survival by a few months [1].

Radiotherapy aims to provide adequate local control, but its role remains controversial in ideal treatment sequence with surgery and improvement in survival [2].

Percutaneous image-guided thermal ablations have been used in different clinical settings, with the aim of achieving the complete ablation of the tumour with reduced invasiveness in comparison with surgical resection. However, few reports have described the clinical application of image-guided thermal ablations in the treatment of recurrent sarcomas [1–10] and the majority of them referred to the treatment of liver, lung or other organs’ metastases. In particular, among the different ablative techniques, percutaneous laser ablation (PLA) seems to represent the ideal technique for treating small lesions in critical structures due to the very small calibre of the applicator and the extreme precision of the ablation area.

In the present paper, we report a case of a patient with recurrent sarcoma successfully treated with the use of PLA.

## Case

A 52-year-old man received the first diagnosis of a monophasic fibrous synovial sarcoma of the left popliteal fossa in 2011, at the age of 45.

In the same year, he had the first surgical intervention, which consisted of the enucleation of a lesion on the thigh. Then, despite two subsequent cycles of chemotherapy (Ifosfamide and Epirubicin), the disease locally progressed, so the oncologists decided to perform preoperative radiotherapy and then a second, more radical surgical intervention the year later.

Until the end of 2014, the patient was stable, with no local or systemic recurrences.

He had to be treated again in 2015 and 2016 for local relapses with limb-sparing surgeries, including also a left femoro-popliteal bypass. After these operations, the disease distantly progressed with a pulmonary metastatic spread. Then, the patient was treated with four high-dose, neoadjuvant chemotherapy cycles (Ifosfamide), unfortunately without a complete response: the lung lesions decreased but did not completely disappear. So, in August 2017, the patient underwent microwave ablation of the four larger lung lesions in order to achieve control of the disease.

**Figure 1.**
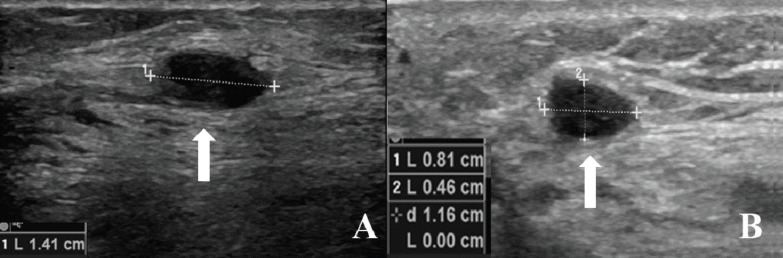
US examination shown in (A): The 14-mm pathologic nodule (arrow) near the scar in the soleus muscle and (B): The 8-mm pathologic nodule (arrow) near the scar of the lateral calf of the gastrocnemius.

Moreover, on 2 October 2017, control magnetic resonance imaging (MRI) of the left leg and knee showed a new local relapse consisting of two pathologic nodules: one of 8 mm near the scar of the lateral calf of the gastrocnemius and one of 14 mm near the scar in the soleus muscle. Both nodules were also visible at ultrasound (US) examination (Figure 1A and B).

Lesion surgical excision was considered not feasible due to previous therapies, and the first proposed treatment option for this recurrence was leg amputation, which the patient refused. Thus, together with the patient’s oncologist and radiation oncologist, the interventional radiologist decided to attempt treatment with PLA under US-guidance in order to avoid a major amputation.

The patient agreed and gave informed consent to the treatment.

## Procedure

Before starting the procedure, 0.5 mg im of atropine and 2 mg iv of midazolam were given to the patient. Then, the patient was turned into the prone position. US examination, performed also with contrast material (contrast-enhanced ultrasound (CEUS)), showed both the lesions in the soft tissue of the posterior sector of the left leg.

**Figure 2.**
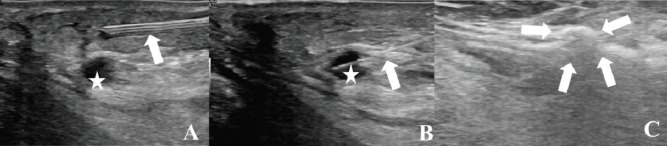
Laser ablation. (A): Injection of local anaesthesia through a small needle (arrow) at the level of the more dorsal lesion (star). (B): A bare-tip, 300 μm in diameter laser fibre, is introduced through a small needle (arrow) into the lesion to be treated (star). (C): The arrows show the gas bubbles produced by the procedure. After another injection of local anaesthesia and hydro-dissection, the medial lesion was treated inserting two parallel 21-gauge Chiba needles and laser fibres. Thermal ablation was performed with one pull-back and a total energy of 3,200 Joule.

Local anaesthesia with Mepivacain diluted in physiological solution was injected at the level of the more dorsal lesion, also achieving a separation of the lesion from the skin (Figure 2A). Under US-guidance, PLA was performed by using a laser unit (Echolaser, Elesta Srl, Florence, Italy). The laser source is a semiconductor diode with a wavelength of 1,064 nm, and a multi-source device enables the use of up to four 300 μm fibres at once. A 21-gauge Chiba needle was placed in the centre of the lesion. Then, a bare-tip, 300 μm in diameter laser fibre was introduced through the needle. Ablation with one pull-back and a total amount of energy of 2,400 Joules was performed at a fixed power of 3 W (Figure 2A and C).

During the procedure, the patient also underwent continuous monitoring of pulse-oximetry, electrocardiography (ECG) and blood pressure.

**Figure 3.**
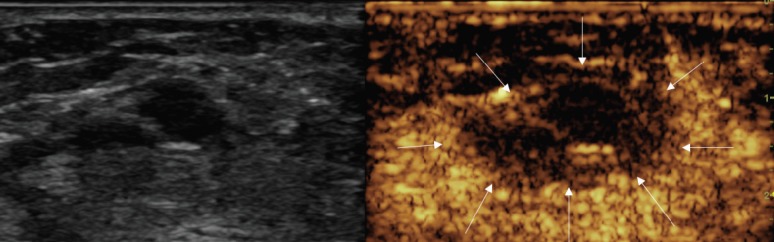
Post-treatment CEUS showing large avascular area, including the 14-mm treated nodule (arrows).

**Figure 4.**
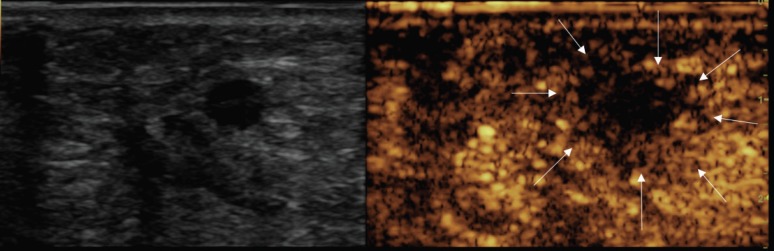
Post-treatment CEUS showing large avascular area, including the 8-mm treated nodule (arrows).

At the end of the procedure, a CEUS showed a good devascularisation of both the treated lesions with a good safety margin. In fact, there was no evidence of residual hyper-enhancing foci, suggesting complete necrosis of both nodules (Figures 3 and 4).

No complications were observed during or after treatment.

MRI study with and without contrast-enhancement was performed within 2 months after laser ablation to evaluate the antitumour effects. The exam showed the complete disappearance of tumour enhancement of both treated nodules, without signs referable to the persistence of the disease or to the appearance of new solid nodules suspected for active disease. This was regarded as complete tumour ablation.

**Figure 5.**
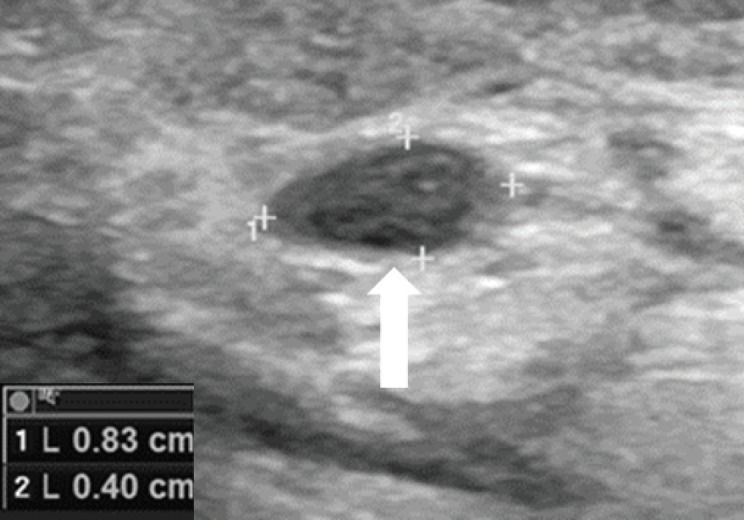
Two months after PLA, the 14-mm nodule appeared smaller (8 × 4 mm) than before the treatment (arrow).

Two months after PLA, CEUS also confirmed the good outcomes of treatment, showing no enhancement of the treated nodules, smaller than before the treatment (Figure 5).

However, CEUS showed the appearance of two new suspected nodules, cranial and lateral compared to the treated ones, the major 8 mm long. These new nodules were confirmed at US examination 6 months after PLA while previously treated lesions were further reduced.

## Discussion

The therapeutic approaches in patients with locally recurrent sarcomas have been largely debated during the last decades [6, 11]: for some authors, the outcome in patients with recurrent extremity soft-tissue sarcomas depends on metastatic disease [12], for others, it relies on the control of the primary tumour [13]. For these reasons, an aggressive approach is warranted in the treatment of local recurrence [6]. Local treatments include surgery and radiotherapy. Percutaneous tumour ablation techniques, especially radiofrequency ablation, have been attempted in few cases to achieve local control of the disease [1–3, 6]. A significant advantage of percutaneous thermal ablation over surgery and radiation includes the repeatability of ablation, as it might be repeated several times in case of recurrence [7]. Moreover, compared with surgery, ablation offers the potential of decreased recovery time, a less invasive procedure, and is often performed in patients deemed not medically fit for surgery [7].

Particularly, as in our case, image-guided thermal ablation offers the possibility to postpone or avoid limb amputation, with a significant advantage for the patients’ quality of life.

To the best of our knowledge, only a few reports have described image-guided ablations in controlling recurrent sarcomas [1–10] and the majority of them referred to the treatment of liver, lung or other organs’ metastases.

In 2014, Yamakado *et al* [3] published a retrospective study about the clinical utility of radiofrequency ablation (RFA) in 52 inoperable patients (two of whom were children) with recurrent bone and soft-tissue sarcomas. In this study, they showed that RFA was a useful and safe therapeutic option in these patients [3].

In 2015, Belfiore *et al* [6] also published a case report about the use of RFA under computed-tomography (CT) guidance to treat a 13 cm recurrent malignant fibrous histiocytoma of the right thigh in an 83-year-old female patient. After ablation, a marked shrinkage of the tumour was obtained. Further local recurrences occurred during follow-up and were safely treated by RFA. At the 6-year follow-up, the patient was still alive and presented a local control of disease, without the metastatic spread of the tumour [6]. Belfiore *et al* [6] reported RFA to be safe, effective and repeatable for soft-tissue sarcoma recurrences, allowing long-term local control of the disease. Even if from these small experiences it is not possible to draw definitive conclusions regarding the role of thermal ablations in soft tissue sarcomas, it seems possible to consider thermal ablations among the toolbox of therapeutic options to be taken into consideration when disease control is the goal of treatment and invasiveness needs to be kept at a minimum.

Jones *et al* [4] aimed to evaluate the efficacy and safety of RFA for both lung and liver metastases in a series of sarcoma patients, demonstrating that it was feasible, effective and well tolerated if the goal of therapy is local disease control. The role of interventional radiology in treating metastatic soft tissue sarcomas is also mentioned in the works of Gronchi *et al* [5] and Olivier *et al* [1]. Moreover, Jones *et al* [4] and Olivier *et al* [1] found RFA most effective to treat lesions ≤ 35 mm in inoperable patients.

We chose to treat our patient using PLA because of the number and the dimensions of the lesions. In fact, PLA has been proposed as the ablation technique of choice in patients with multiple and small tumours or for cases with a high-risk location of the lesion to be treated, such as lesions in the neck or renal tumour at high bleeding risk [14–17]. Moreover, the diameter of PLA needles is smaller than RFA electrodes and microwave ablation antennas, making PLA safer and more suitable to ablate lesions with at-risk location and/or difficult to be reached [17–19].

Moreover, both lesions were visible at US examinations, so we decided to treat them under US guidance; as mentioned above, other authors opted for an ablation treatment under CT guidance [3, 6].

In case of poor visibility at US, the use of CEUS has been reported to be extremely helpful to enhance the conspicuity of a lesion in order to better depict it and to enhance the accuracy of image-guided targeting [20].

Moreover, immediate post-procedural CEUS is strongly recommended to assess the completeness of the ablation treatment and to guide the immediate re-treatment in case of residual viable tissue [21–23].

Another option that can help interventional radiologists in cases of lesions being invisible at US and not completely depictable with CEUS is the use of virtual navigations systems and fusion imaging, which has been reported to be a safe, feasible and effective strategy [24–26]. Finally, hydrodissection can be used to achieve a safe distance between the target lesion and surrounding critical structures, thus making it possible to also perform ablation in cases with difficult location [14, 24]. In the presented case, we used hydrodissection to achieve a safe distance between the lesion and the superficial skin and deep structures, such as surgical bypass. This allowed us to perform a complete ablation without damaging surrounding critical structures.

## Conclusion

In conclusion, from our very preliminary experience, PLA seems to represent a feasible, tolerable and effective technique for the local control of recurrent sarcomas. Further studies on larger series are needed to confirm these preliminary results. PLA can be considered to be within the therapeutic toolbox for patients with sarcoma when the principal aim is to achieve local disease control by minimising treatment invasiveness.

## Conflicts of interest

G Mauri is a consultant for Elesta SrL. All of the other authors have no conflicts of interest.

## Funding statement

The authors received no specific funding for this work.
